# Publisher Correction: HIV-1 replication complexes accumulate in nuclear speckles and integrate into speckle-associated genomic domains

**DOI:** 10.1038/s41467-020-20152-w

**Published:** 2020-11-26

**Authors:** Ashwanth C. Francis, Mariana Marin, Parmit K. Singh, Vasudevan Achuthan, Mathew J. Prellberg, Kristina Palermino-Rowland, Shuiyun Lan, Philip R. Tedbury, Stefan G. Sarafianos, Alan N. Engelman, Gregory B. Melikyan

**Affiliations:** 1 Division of Infectious Diseases, Department of Pediatrics, Emory University School of Medicine, Atlanta, GA 30322 USA; 2 Department of Cancer Immunology and Virology, Dana-Farber Cancer Institute, Boston, MA 02115 USA; 3 Department of Medicine, Harvard Medical School, Boston, MA 02115 USA; 4 Laboratory of Biochemical Pharmacology, Department of Pediatrics, Emory University School of Medicine, Atlanta, GA 30322 USA; 5 Children’s Healthcare of Atlanta, Atlanta, GA 30322 USA

**Keywords:** Microbiology, Virology

Correction to: *Nature Communications* 10.1038/s41467-020-17256-8, published online 14 July 2020.

The original version of this Article contained several errors in Fig. 7. In Fig. 7a, the PF74 concentration for the middle panel was incorrectly labelled as ‘25’, rather than the correct ‘2.5’. In Fig. 7b, the PF74 concentration for the blue series was incorrectly labelled as ‘25’, rather than the correct ‘2.5’. In Fig. 7c, the PF74 concentration for the third panel from the top was incorrectly labelled as ‘25’, rather than the correct ‘2.5’. In Figs. 7d and e, the PF74 concentrations for the green series were incorrectly labelled as ‘2.5’, rather than the correct ‘25’. These errors have all been corrected in both the PDF and HTML versions of the Article.

