# Supplementary material for: HIV-1 replication complexes accumulate in nuclear speckles and integrate into speckle-associated genomic domains
Source: Nat Commun. 2020 Jul 14;11:3505. doi: 10.1038/s41467-020-17256-8 (PMC7360574; doi:10.1038/s41467-020-17256-8)
Supplement: Supplementary file 1 — Supplementary Information [file 41467_2020_17256_MOESM1_ESM.pdf]

HIV-1 replication complexes accumulate in nuclear speckles and integrate into speckle-associated genomic domains

Ashwanth C. Francis et al.

Supplemental Information

1. **Supplementary Figures and Legends**
2. **Supplementary Table 1.**

1. Supplementary Figures and Legends

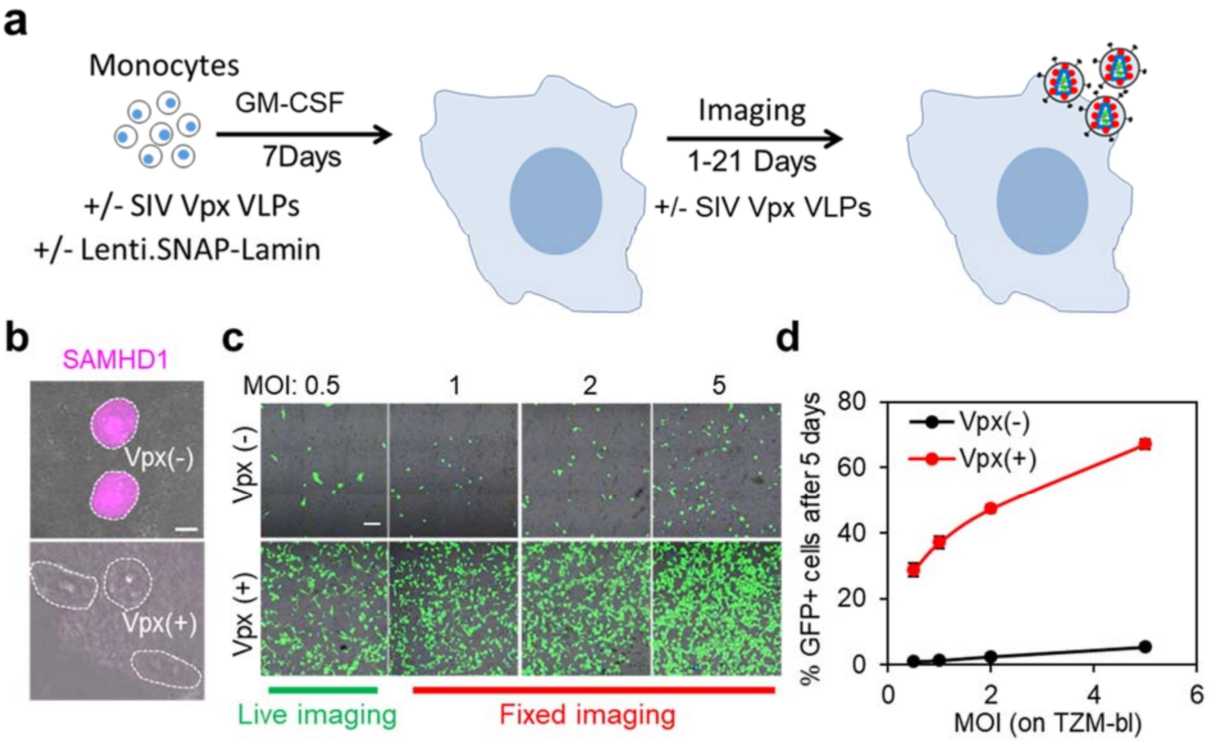

Supplementary Fig. 1. Preparation of human MDMs and characterization of HIV-1 infection *(Related to Fig. 1)*. (**a**) Schematic illustration of MDM preparation. (**b**) Treatment of monocytes with Vpx(+) results in efficient depletion of SAMHD1 in MDMs. Vpx(+) or untreated (Vpx(-)) monocytes were differentiated as in (**a**). MDMs were fixed at day 17 post-GM-CSF removal and analyzed for SAMHD1 expression by immunostaining. Representative images (overlay of fluorescence and Differential Interference Contrast (DIC) channels) of untreated MDMs (top) and Vpx(+) treated (bottom) MDMs are shown. Scale bar 5 µm. (**c, d**) HIV-1 infection of MDMs progresses for several days. MDMs treated with or without SIV Vpx were infected with VSV-G pseudotyped HIVeGFP fluorescently tagged with INmNG at different MOIs determined in TZM-bl cells. MDMs were examined for eGFP expression at 5 d post-infection using a confocal microscope. Images (**c**) and quantification of eGFP expression (**d**) from a representative experiment from 2 donors are shown. Scale bar 200 µm. Error bars are mean values +/-SEM from 4 fields of view. Images in (b) and (c) are representative of >50 nuclei and 27 fields of view, respectively, from 3 independent experiments/donors.

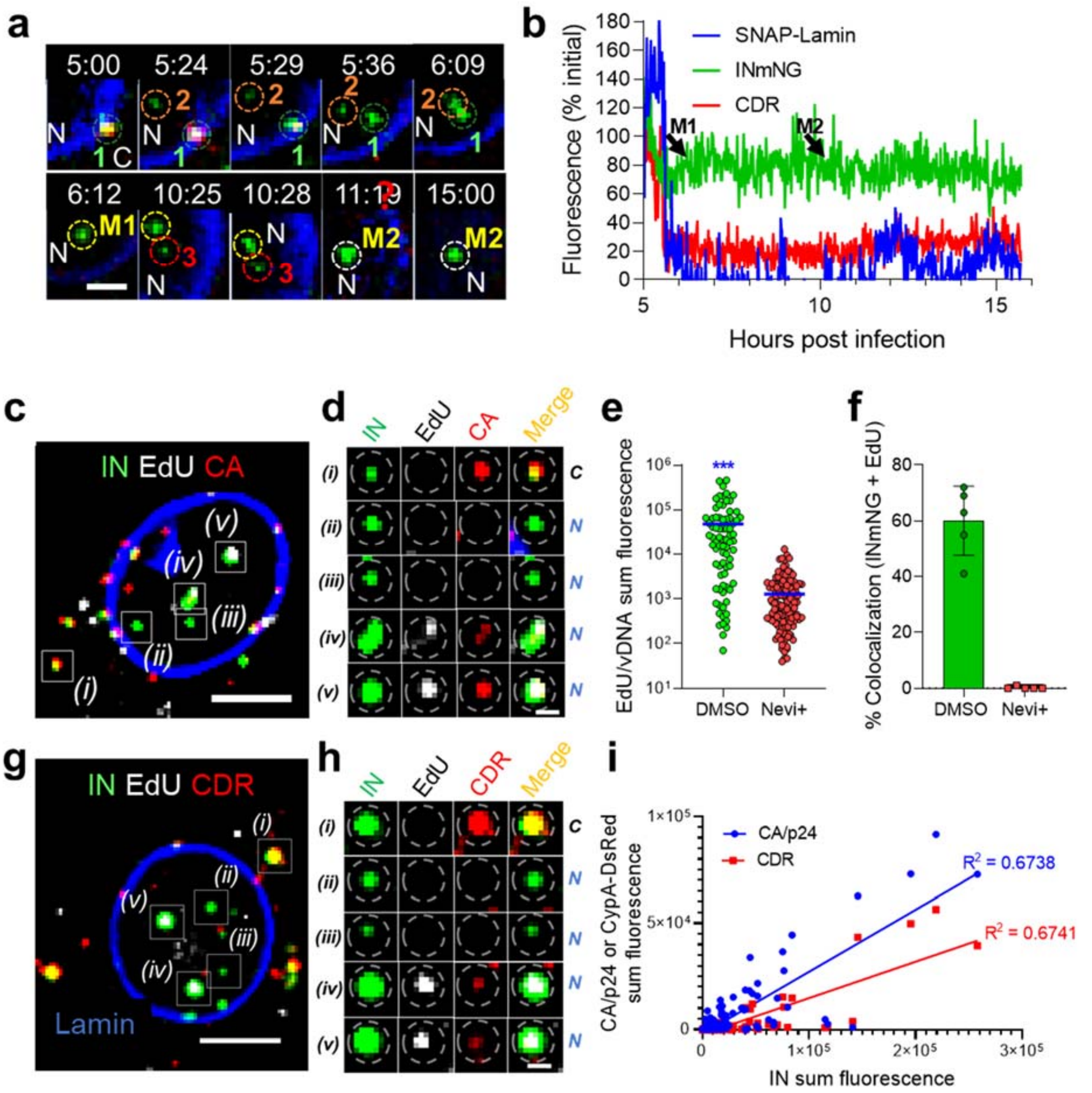

Supplementary Fig. 2. HIV-1 VRCs uncoat at the nuclear membrane, enter the nucleus, and merge with other complexes *(Related to Fig. 1)*. (**a, b**) Nuclear import was imaged in Vpx(+) treated MDMs expressing SNAP-Lamin (blue) (as in Fig. 1**a, b**) infected with VSV-G pseudotyped HIVeGFP co-labeled with INmNG and CypA-DsRed (CDR). Time lapse images were collected 90s/volume from 0.5 – 16 hpi. (**a**) Images (single Z-slices) with time stamps showing three IN-labeled (green) particles merging after entry into the nucleus. An INmNG/CDR co-labeled single core (1, green dashed circle) docked at the nuclear envelope lost CDR and entered into the nucleus. A second IN complex (2, orange dashed circle) entered the nucleus and merged with the first complex (M1, yellow dashed circle at 6h 12m). After 4 h, a 3rd IN complex entered the nucleus (3, red dashed circle at 10h 25m), trafficked and merged with M1 (M2, white dashed circle). The three complexes remained together till the end of the movie. (**b**) Mean fluorescence intensities of INmNG (green) and CDR (red) of the single virus (1, green dashed circle) in panel (**a**). Arrows

mark the time of merger of complexes 1 and 2 (M1) and 1, 2 and 3 (M2). (**c-i**) MDMs (Vpx(+) treated) were infected with VSV-G pseudotyped pR9ΔEnv labeled with INmNG alone (**c-f** and **i**) or INmNG and CypA-DsRed (CDR) (**g-i**) in the presence of 5 μM EdU (**c-h**). MDMs were fixed at 24 hpi and stained for EdU/vDNA (**c-h)**, CA (**c-f**) and lamin (**c-i**). (**c, g**). A 2 μm-thick central Z-projection of the MDM nucleus showing various sizes and staining patterns for cytoplasmic *(i)* or nuclear IN puncta *(ii – v)* marked in white boxes. (**d**, **h**) Blow-up of boxes in (**c,g**) showing the CA (**d**) or CDR (**h**) signals (red) and EdU signal (white, **d, h**) colocalized with IN (green) puncta. (**e, f**) Reverse transcription inhibitor Nevirapine (Nevi+, 10 μM) blocks the incorporation of EdU into IN complexes. Nuclear IN puncta were detected and the associated EdU sum fluorescence is plotted (**e**) Colocalization of EdU/vDNA puncta (EdU/vDNA puncta was detected as low as 0.02/nucleus) with INmNG puncta is shown in (f). (**i**) Correlation between IN-colocalized CA (c, d) and CDR (g, h) signals with respective fluorescence intensity of INmNG complexes. Pearson's correlation for CA (shown in blue) and CDR (shown in red) signals with INmNG signal. Scale bars are 2 μm (a), 5 μm (c and g) and 0.5 μm (d, h). Error bars in (f) are mean values +/-SEM (individual data points are shown); in (e) median values at 95% CI for n>50 nuclei from 3-independent donors are shown.

a

Treatment duration

Drug	1hr Did it break clusters?	24hr Did it break clusters?	Target
PF74 (25μM) (Shi et al., 2011)	No	No	CA
CA-I (100μM) (Chen et al., 2016)	No	No	CA
TNFα (1μg/ml)	No	No	Latency reversal / Cell-activation
5,6-Dichlorobenzimidazole 1-β-D-ribofuranoside (DBR) (50μg/ml) (Kim et al., 2019)	No	No	RNA-PolII
α-Amanitin (50μg/ml) (Kim et al., 2019; Lamond and Spector 2003)	No	No	RNA-PolII
1-6 Hexanediol (10%) (Elbaum-Garfinkle, 2019; Lu et al., 2018; Sabari et al., 2018)	0.5hr No	-	Liquid-liquid phase separation

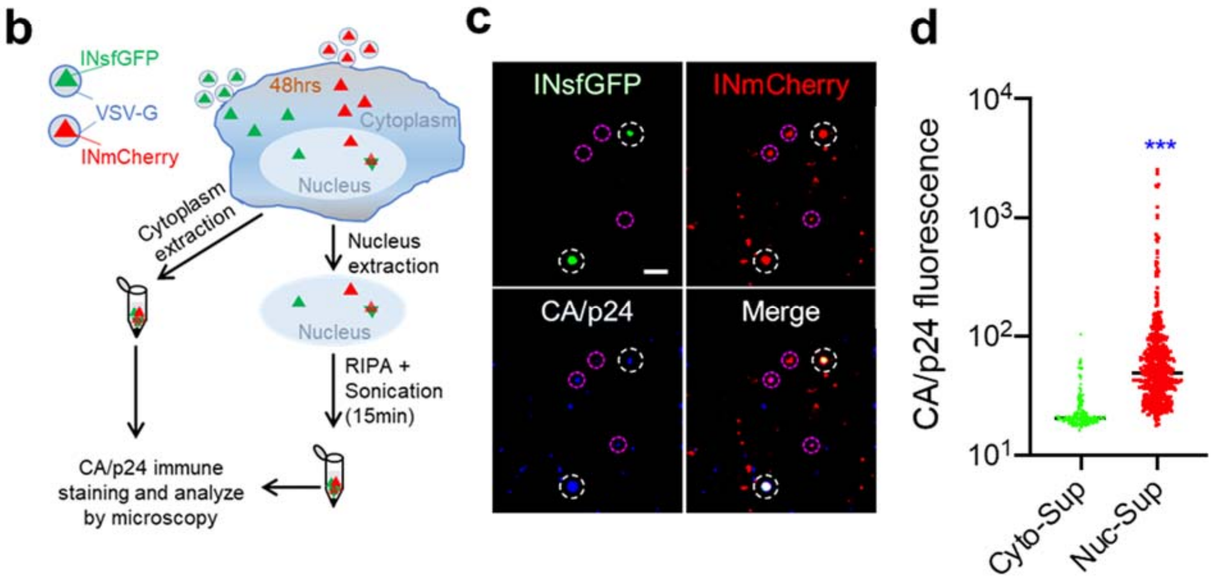

Supplementary Fig. 3. Nuclear HIV-1 VRC clusters are highly stable *(Related to Figs. 1 and 2)*. MDMs were co-infected with viruses labeled with either INmNG or INmCherry for 48 h to allow nuclear cluster formation. (**a**) A table summarizing the lack of effect of different drug treatments on VRC clusters. Cells were treated with the indicated compounds and co-mobility of INmNG and INmCherry complexes was visualized for 1 h by live-cell imaging or incubated for additional 24 h and re-examined by confocal microscopy in fixed cells. The lack of effect on colocalization of INmNG and INmCherry is marked as 'No' effect. The 1-6 Hexanediol (10%) treatment resulted in morphological changes and cell death after 60 min, without affecting colocalization of INmNG+INmCherry in VRC clusters. (**b**) Schematic of the experimental design for extracting nuclear VRC clusters. The cytosolic fraction was extracted by a brief treatment with buffer containing 0.05% NP40. The remaining nuclear fraction was lysed with RIPA buffer and sonicated for 15 min to shear DNA and RNA, followed by a brief centrifugation step to remove cellular debris. The supernatant was added to a poly-L-lysine coated cover glass, immuno-labeled for CA, and examined by fluorescence microscopy. (**c**) Representative images from 3 independent experiments show two-color VRC clusters (green and red) co-immunostained for CA (blue). Scale bar 2 µm. (**d**) Analysis of coverslip-adhered IN complexes from cytosolic and nuclear soluble fractions. Distribution of mean fluorescence intensities of CA. Error bars are median values +/- SEM at 95% CI from 3 independent experiments. p values in (d) are from non-parametric Mann-Whitney Rank Sum test (ns, $p>0.05$, ***, $p<0.001$).

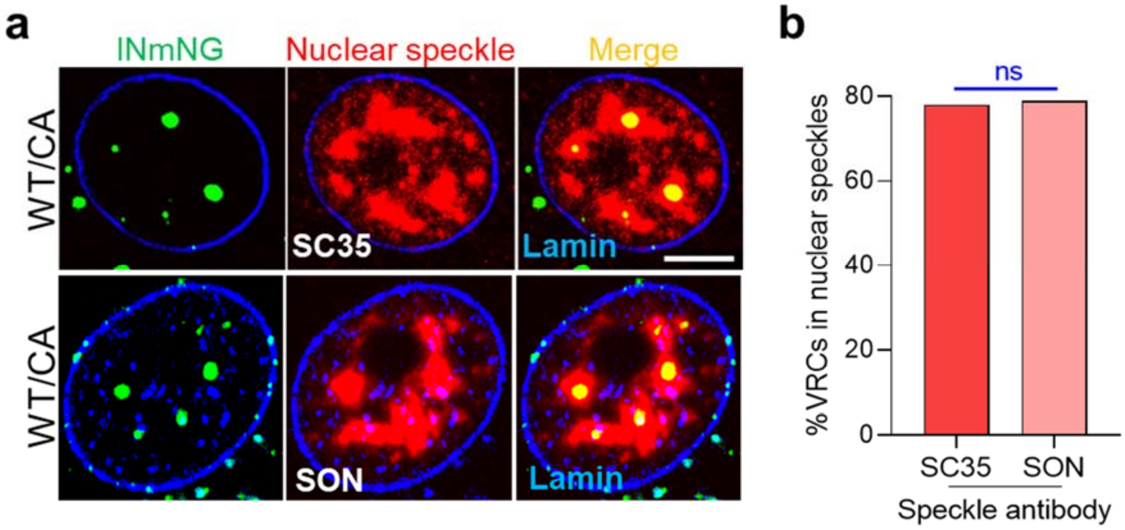

Supplementary Fig. 4. HIV-1 VRCs are compartmentalized in NSs of MDMs *(Related to Fig. 2)*. (**a, b**) VSV-G pseudotyped HIVeGFP labeled with INmNG was used to infect MDMs at MOI of 5 for 24 h. Cells were fixed and immunostained for NSs using anti-SC35 or anti-SON antibodies. Single Z-plane images (**a**) and colocalization analysis of nuclear INmNG puncta with SC35 and SON labeled NS-compartments (**b**) are shown. Scale bar in (a) is 5μm. Mean values are shown in (b) from n>30 nuclei (96 and 108 nuclear IN-complexes were analyzed) from a single experiment. Statistical significance in (b) was determined by two-tailed Student's t-test, ns (p=0.5850).

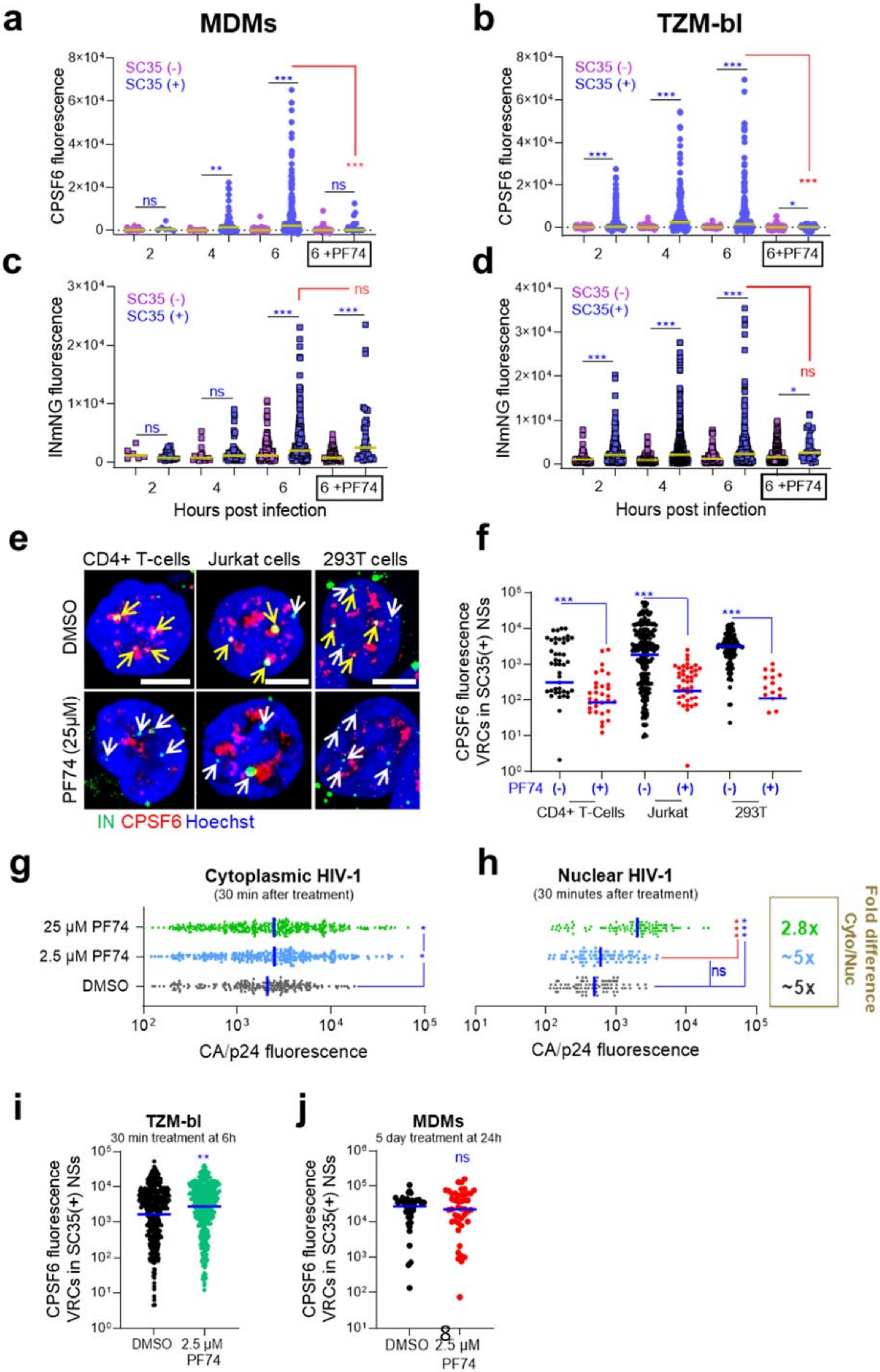
a
MDMs
b
TZM-bl
SC35 (-)
SC35 (+)
CPSF6 fluorescence
INmNG fluorescence
c
d
Hours post infection
2
4
6
6 +PF74
ns
e
CD4+ T-cells
Jurkat cells
293T cells
DMSO
PF74 (25μM)
IN CPSF6 Hoechst
f
CPSF6 fluorescence VRCs in SC35(+) NSs
PF74 (-) (+)
CD4+ T-Cells
Jurkat
293T
g
Cytoplasmic HIV-1
(30 min after treatment)
25 μM PF74
2.5 μM PF74
DMSO
CA/p24 fluorescence
h
Nuclear HIV-1
(30 minutes after treatment)
2.8x
~5x
~5x
Fold difference Cyto/Nuc
i
TZM-bl
30 min treatment at 6h
DMSO
2.5 μM PF74
j
MDMs
5 day treatment at 24h

Supplementary Fig. 5. CPFS6 recruitment stabilizes HIV-1 VRC association with NSs *(Related to Figs. 4-6).* VSV-G pseudotyped HIVeGFP labeled with INmNG was used to infect MDMs, TZM-bl and HEK293T cells by spinoculation; Jurkat and primary CD4+ T-cells were infected in suspension. Cells were fixed at varied time points (**a-d**) or at 6 hpi (**e-h**) and immunostained for CPSF6 and NSs (SC35). Shown are CPSF6 (**a, b**) or INmNG (**c, d**) intensities associated with nuclear IN puncta residing inside (SC35(+)) or outside (SC35(-)) NSs in MDMs (**a, c**) or TZM-bl cells (**b, d**) (see also Fig. 4 **a-d**). (**e**) Maximum intensity projections of the central Z-slices (1 μm) from primary CD4+ T-cells, Jurkat, and HEK293T cells showing CPSF6 association with NS-localized IN puncta (top panels) and the loss of CPSF6 signal and NS localization during a 30 min 25 μM PF74 treatment at 6 hpi (bottom panel). Yellow and white arrows point to IN puncta inside and outside NSs, respectively. Scale bar is 5 μm. (**f**) A 30-min treatment with 25 μM PF74 displaces CPSF6 from NS-localized IN spots. CPSF6 intensities associated with NS-localized IN puncta in the absence (-) or presence (+) of 25 μM PF74 in indicated cells are shown. (**g, h**) A brief treatment with PF74 increases CA signal in cellular VRCs. After live cell imaging (Fig. 5 e, f), cells were fixed and stained for CA. The CA signal associated with INmNG puncta in the cytoplasm (**g**) and nucleus (**h**) is shown. Note that only 25 μM PF74 treatment resulted in significant increase in CA detection in the nucleus. The fold-difference in mean CA/p24 signals after HIV-1 nuclear import was determined by dividing mean CA intensities in cytoplasmic IN puncta by those associated with nuclear IN puncta. Average fold-differences from 3 independent experiment are shown on the right of panel (h). Effect of 2.5 μM PF74 on the nuclear VRC-associated CPSF6 signal was measured in TZM-bl cells treated for 30 min at 6 hpi (**i**) or in MDMs treated for 5 d starting from 24 hpi (**j**). Yellow lines in (a-d) and blue lines in (f-j) represent median values at 95% CI from 3 independent experiments.

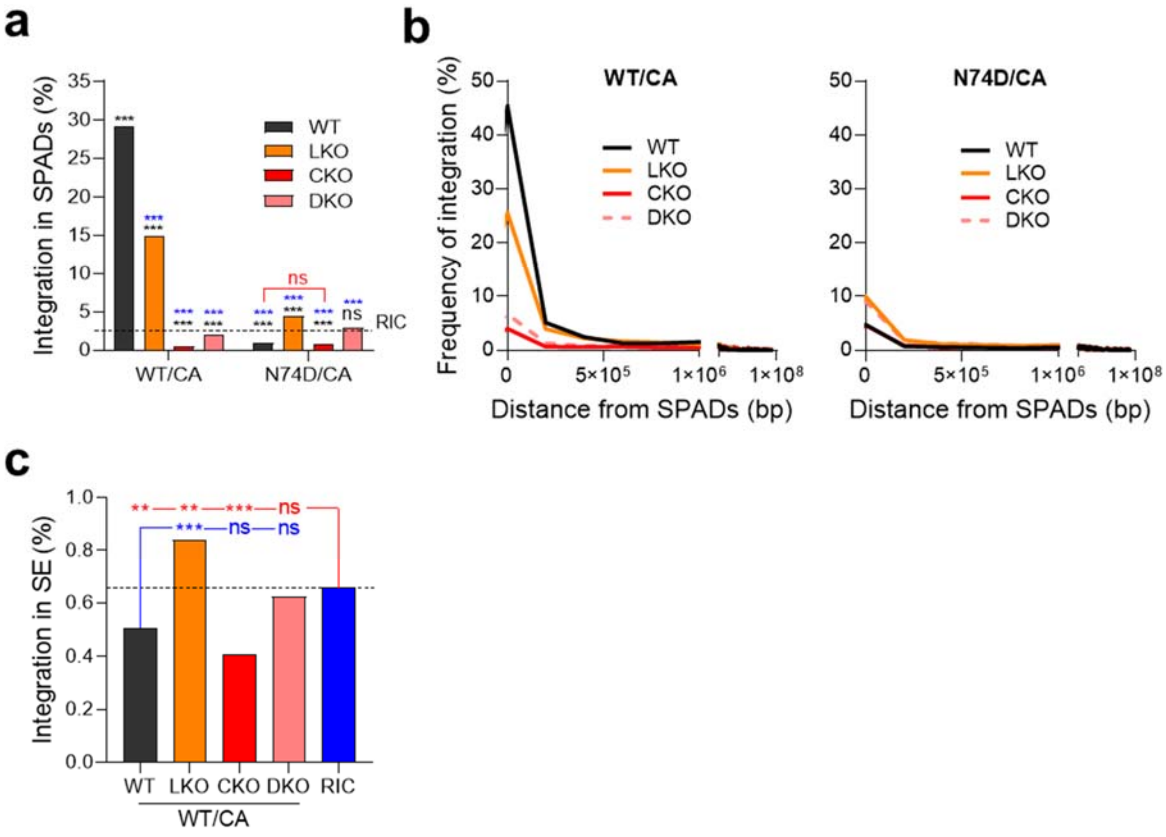

Supplementary Fig. 6. CPSF6-dependent integration targeting of SPADs *(Related to Fig. 7A-H)*. (**a, b**) Analysis of HIV-1 WT/CA and N74D mutant CA (N74D/CA) integration preferences into SPAD regions in HEK293T cells (WT) and HEK293T cells lacking CPSF6 (CKO), LEDGF/p75 (LKO), or both proteins (DKO). Percent of HIV-1 integrations occurring within SPAD regions (**a**) and the frequency distribution of all integration sites as a function of their relative distance from closest SPADs (**b**). (**c**) Bulk integration sites in SEs for the cell types indicated in (**a**). P values versus matched WT conditions (blue asterisks) or RIC (black asterisks) were calculated by Fisher's exact test (ns, > 0.05; ***, < 0.0001; in A, N74D infection of WT cells was compared to WT HIV-1 infection of WT cells). Dashed lines in (**a-b**), RIC (2.8% SPAD targeting).

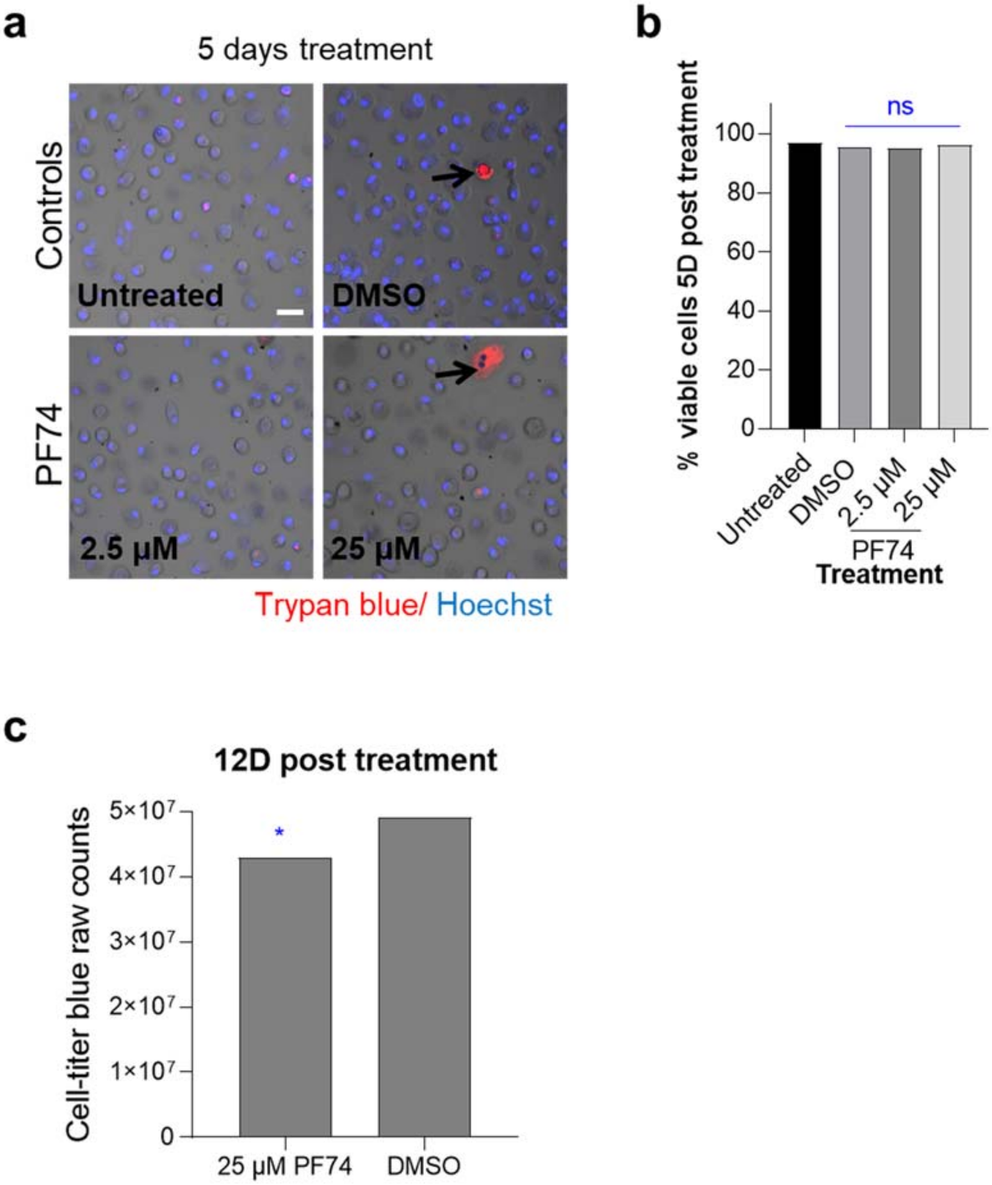

Supplementary Fig. 7. Cell viability assay *(Related to Figs. 6)*. (**a, b**) The viability of MDMs treated with the indicated concentrations of PF74 for 5 days was measured by trypan blue staining (excited using 561 nm laser line and emission collected between 562-625 nm with a 20x objective using a Zeiss LSM 880 microscope. Cell nuclei was stained with Hoechst-33342 and the fraction of viable cells negative for trypan blue staining was visualized (**a**). Black arrow in DMSO and 25 µM PF74 treated cells indicate a dead cell stained with trypan blue and negative for nuclear stain. (**b**) Quantification of fraction of viable cells negative for trypan blue. (**c**) A similar result was obtained for cell-viability after a 12-day treatment with 25 µM PF74 using an alternative cell viability measurement method Cell-Titer Blue assay. Mean values are shown in (b) from 16 fields of view from 2-independent experiments and in (c) from a single triplicate experiment. Statistical significance relative to DMSO control in (b, c) was determined by two-tailed Student's t-test, ns in (b) $p>0.12$ and * in (c) $p=0.0279$.

Supplementary Table 1. CPSF6-dependent integration targeting of SPADs and SE regions *(Related to Fig. 7G, H)*. RIGs identified in at least three of four studied cell types (HEK293T, HOS, MDM, and primary CD4+ T cells) are shown with their respective gene IDs and distances to the nearest SPAD and SE in bp. A total of 46 CPSF6(+) RIGs and 30 CPSF6(-) RIGs were identified. Also shown are mean distances across RIGs for the indicated genomic annotation.
