# Supplementary material for: HIV-1 replication complexes accumulate in nuclear speckles and integrate into speckle-associated genomic domains
Source: Nat Commun. 2020 Jul 14;11:3505. doi: 10.1038/s41467-020-17256-8 (PMC7360574; doi:10.1038/s41467-020-17256-8)
Supplement: Supplementary file 3 — Reporting Summary [file 41467_2020_17256_MOESM3_ESM.pdf]

nature research

Corresponding author(s): Gregory Melikyan and Ashwanth Francis

Last updated by author(s): Jun 17, 2020

Reporting Summary

Nature Research wishes to improve the reproducibility of the work that we publish. This form provides structure for consistency and transparency in reporting. For further information on Nature Research policies, see our Editorial Policies and the Editorial Policy Checklist.

Statistics

For all statistical analyses, confirm that the following items are present in the figure legend, table legend, main text, or Methods section.

n/a	Confirmed	
☐	☒	The exact sample size (*n*) for each experimental group/condition, given as a discrete number and unit of measurement
☐	☒	A statement on whether measurements were taken from distinct samples or whether the same sample was measured repeatedly
☐	☒	The statistical test(s) used AND whether they are one- or two-sided<br>*Only common tests should be described solely by name; describe more complex techniques in the Methods section.*
☒	☐	A description of all covariates tested
☒	☐	A description of any assumptions or corrections, such as tests of normality and adjustment for multiple comparisons
☐	☒	A full description of the statistical parameters including central tendency (e.g. means) or other basic estimates (e.g. regression coefficient) AND variation (e.g. standard deviation) or associated estimates of uncertainty (e.g. confidence intervals)
☐	☒	For null hypothesis testing, the test statistic (e.g. F, t, r) with confidence intervals, effect sizes, degrees of freedom and P value noted<br>*Give P values as exact values whenever suitable.*
☒	☐	For Bayesian analysis, information on the choice of priors and Markov chain Monte Carlo settings
☒	☐	For hierarchical and complex designs, identification of the appropriate level for tests and full reporting of outcomes
☒	☐	Estimates of effect sizes (e.g. Cohen's d, Pearson's r), indicating how they were calculated

Our web collection on statistics for biologists contains articles on many of the points above.

Software and code

Policy information about availability of computer code

Data collection	Carl Zeiss MicroImaging Zen software. V2.3 SP1
Data analysis	ICY image analysis software: V2.0.1.0; V2.0.2.0 and V2.0.3.0

For manuscripts utilizing custom algorithms or software that are central to the research but not yet described in published literature, software must be made available to editors and reviewers. We strongly encourage code deposition in a community repository (e.g. GitHub). See the Nature Research guidelines for submitting code & software for further information.

Data

Policy information about availability of data

All manuscripts must include a data availability statement. This statement should provide the following information, where applicable:

- Accession codes, unique identifiers, or web links for publicly available datasets
- A list of figures that have associated raw data
- A description of any restrictions on data availability

Data availability statement is included in the manuscript. For SPAD analysis, raw sequences were aligned to hg19 using Bowtie2 v2.3.4.3 and Samtools v1.3.1 was used to create Bam files. The ranking of TSA-Seq reads to > 95th percentile was done using RStudio v1.2.5001. The dbSUPER database was accessed via https://asntech.org/dbsuper/. For integration sites, raw sequences were aligned to hg19 using BLAT v35 and HISAT2 v2.1.0. The Bedtool intersect command was performed via Bedtools v2.27.1. Python v3.7.4 and its module scipy.stats was used for to perform Fisher's exact test.

Field-specific reporting

Please select the one below that is the best fit for your research. If you are not sure, read the appropriate sections before making your selection.

☒ Life sciences ☐ Behavioural & social sciences ☐ Ecological, evolutionary & environmental sciences

For a reference copy of the document with all sections, see nature.com/documents/nr-reporting-summary-flat.pdf

Life sciences study design

All studies must disclose on these points even when the disclosure is negative.

Sample size	No sample size calculations were performed. The sample size for live-cell MDM experiments involving single particle tracking was determined by the fraction of particles entering the nucleus or losing CPSF6 in the nucleus in response to PF74 addition. For fixed cell experiments, the sample size typically exceeded n=l00 and was chosen to achieve acceptable confidence intervals.
Data exclusions	For live-cell nuclear import experiments, viral complexes entering the nucleus from poorly defined lamin boundary were excluded from analysis. None of the data related to the current version of the paper have been excluded. The statistical significance was determined at 95% confidence intervals, but all data points are shown in the respective graphs. This type of analysis was performed in Fig. 1d, f; Fig. 3b-e; Fig.4 c, d; Fig. 7d; Suppl. Figs. 2e, f; 3d; 5a-d, f-j.
Replication	We ensured reproducibility of our findings using biological and technical replicates. Unless indicated otherwise, all experiments were reproduced using at least 3 independent biological replicates.
Randomization	The design and development of the experimental strategies was done by the same investigator who also performed the experiments. Therefore, randomization was not possible. Fluorescence microscopy data collection was performed using randomly selected 9-25 neighboring fields of view. Software-based analyses of fluorescence intensities was randomized (blinded to the operator).
Blinding	For initial image annotation, investigators were blinded. Blinded data collection was not possible, since the same investigator was involved in experimental design. Image analysis was double-blinded to the technical operator of the image analysis station.

Reporting for specific materials, systems and methods

We require information from authors about some types of materials, experimental systems and methods used in many studies. Here, indicate whether each material, system or method listed is relevant to your study. If you are not sure if a list item applies to your research, read the appropriate section before selecting a response.

Materials & experimental systems

n/a	Involved in the study	
☐	☒	Antibodies
☐	☒	Eukaryotic cell lines
☒	☐	Palaeontology and archaeology
☒	☐	Animals and other organisms
☒	☐	Human research participants
☒	☐	Clinical data
☒	☐	Dual use research of concern

Methods

n/a	Involved in the study	
☒	☐	ChIP-seq
☒	☐	Flow cytometry
☒	☐	MRI-based neuroimaging

Antibodies

Antibodies used	There was an error in the previous version of the manuscript. The correct antibodies are: Donkey anti-rabbit-AlexaFluor405 ((#ab175651) or Goat anti-rabbit AF568 (Molecular probes, Thermo Scientific; cat# A11011). These are now included in the Methods section.<br><br>Rabbit Anti-SON (polyclonal IgG) was from AtlasAntibodies (#HPA031755)<br><br>Mouse anti-tubulin antibody was not used in the paper and is therefore removed from the current version. Antibody dilutions are specified in the Methods section. Mouse monoclonal primary antibody against CDK9 pS175 was a kind gift from Jonathan Karn (Case Western University) and was developed and validated in the Karn lab.<br>Mouse monoclonal anti-p24 antibody AG3.0 (Cat# 4121) was obtained from the NIH AIDS Reagent Program, Division of AIDS, NIAID, NIH: Anti-HIV-1 p24 Monoclonal (AG3.0) from Dr. Jonathan Allan. “Simm M, Shahubuddin M, Chao W, Allan JS, Volsky DJ. Aberrant Gag protein composition of a human immunodeficiency virus type 1 vif mutant produced in primary lymphocytes. J Virol 69:4582-4586, 1995.”
Validation	Primary antibodies were verified by comparing immunofluorescence images to that of published manufacturers data and websites. Secondary antibodies showed minimal cross-reactivity with primary antibodies from different species. The distinct localization of two

different proteins in co-immunolabeling experiments (Lamin/SC35; Lamin/CPSF6; SC35/CPSF6; CA/CPSF6; CA/SC35; Lamin/CycT1; Lamin/pS175; etc) further attests to the specificity of primary and secondary antibodies.

Eukaryotic cell lines

Policy information about cell lines

Cell line source(s)	293T/17 were from ATCC, TZM-bl cells were from the NIH AIDS Reagent Program, Jurkat cells were obtained from Dr. Jeremy Luban (University of Massachusetts). The primary human MDMs and CD4+ T-cells were derived from PBMCs isolated from de-identified healthy donors.<br>Jurkat cell-lines were a kind gift from Drs. D. Braaten and J. Luban, and originated from the NIH AIDS reagent program. Data obtained with HOS cell lines are not used in the current version of the manuscript and are therefore removed.
Authentication	Commercially obtained cell lines were authenticated by the vendors. MDMs were purified and activated according to validated protocols.
Mycoplasma contamination	Cell lines were tested for mycoplasma contamination and found to be negative for mycoplasma.
Commonly misidentified lines (See ICLAC register)	No commonly misidentified cell lines were used in this study.

April 2020
