# Supplementary material for: HIV-1 replication complexes accumulate in nuclear speckles and integrate into speckle-associated genomic domains
Source: Nat Commun. 2020 Jul 14;11:3505. doi: 10.1038/s41467-020-17256-8 (PMC7360574; doi:10.1038/s41467-020-17256-8)
Supplement: Supplementary file 4 — Description of Additional Supplementary Files [file 41467_2020_17256_MOESM4_ESM.pdf]

Description of Additional Supplementary Files

File Name: Supplementary Movie 1
Description: **Merger of VRCs in the MDM nucleus (Related to Fig. 1a,b).** MDMs with SNAP-Lamin (blue) labeled nuclear envelope were infected with VSV-G pseudotyped HIVeGFP virus co-labeled with INmNG (green, VRC marker) and CDR (red, CA marker). Movie shows uncoating, loss of CA/CDR signal-from a VRC docked at the nuclear envelope followed by nuclear entry. A second VRC enters the nucleus (1h 15m 48s) from a different location. The two VRCs traffic towards each other and merge. Video is related to Fig. 1A, B. The single particle trajectory (+/- 5 frames) was overlaid onto the movie using ICY software. Movie begins at 30 min post-infection and is a maximum intensity projection of 1µm Z-slices. Scale bar 10 µm.

File Name: Supplementary Movie 2
Description: **Merger of VRCs in the MDM nucleus (Related to Fig. 1 and Suppl. Fig. 2a,b).** MDMs were infected with VSV-G pseudotyped HIVeGFP virus colabeled with INmNG (green, VRC marker) and CDR (red, CA marker). Movie shows docking of a CA/CDR containing VRC at the nuclear envelope (labeled with SNAP-Lamin, blue) followed by uncoating (loss of CA/CDR) and transport to a second VRC present in the nucleus, where they cluster. At a later time point a 3rd VRC enters the nucleus (white arrowhead) and merges with the preexisting VRC cluster. The single particle trajectory (+/- 5 frames) was overlaid onto the movie on ICY software. Movie begins at 5 hpi and is a maximum intensity projection of 1µm Zslices. Scale bar 10 µm.

File Name: Supplementary Movie 3
Description: **Nuclear HIV-1 clusters are immobile (Related to Fig. 1c-f).** MDMs were infected for 48 h with two preparations of VSV-G pseudotyped HIV-1 virus fluorescently labeled with either INmNG (green) or INmCherry (red). Movie shows highly restricted motion of merged nuclear VRC clusters (INmNG+INmCherry) in the nucleus, which is not displaced by treatment with PF74 (Ex-1) or 3% Hexanediol (Ex-2). Video is related to Suppl. Fig. 3. The single particle trajectory (+/- 5 frames) was overlaid onto the movie on ICY software. Movie begins at 72 hpi and is a maximum intensity projection of 1µm Z-slices. Diffused green and red signals in the cytoplasm are a result of cellular auto fluorescence. Scale bar 10 µm.

File Name: Supplementary Movie 4
Description: **CPSF6 quickly diffuses in the nucleus (Related to Fig. 5a, b).** Movie shows the mobility of PA-CPSF6 after photo-activation (green polygon) into the non- 3 activated region of the TZM-bl cell nucleus (red polygon in example-1 and white polygon in example-2) within seconds after illumination with a 405 nm laser. Images were acquired every 500ms at a single Z-plane following photo-activation. A zoomed-in view and its respective full view (bottom corner) is shown in Example-1. PA-CPSF6 intensity is shown as a heatmap from high (red) to low (blue). Scale bar is 5 µm.

File Name: Supplementary Movie 5
Description: **PA-CPSF6 is immobile at the location of VRCs in TZM-bl nucleus (Related to Fig. 5c, d).** Movie shows the photo-activation of PA-CPSF6 (red) in different locations (colored boxes). The photo-activated PA-CPSF6 remains at the location of VRCs (marked by white arrowheads) while PA-CPSF6 redistributes in seconds at control locations away from VRCs (blue and magenta boxes). The green fuzzy green signal in the cytoplasm (perinuclear region) is auto-fluorescence. TZM-bl cells infected with INmCherry labeled HIV-1 were imaged starting at 4 hpi. The movie is a maximum intensity projection of 1 µm Z-slices. Scale bar is 5 µm.

File Name: Supplementary Movie 6
Description: **Photo-activated PA-CPSF6 is stably bound to VRCs in TZM-bl nucleus (Related to Fig. 5c, d).** Movie shows co-trafficking of photo-activated PA-CPSF6 (red) with INmCherry-labeled VRCs (green, marked by white arrowheads) for over 2.5 h at which point, the application of 25 µM PF74 results in displacement of PA-CPSF6 from VRCs. The green fuzzy signal in the cytoplasm (perinuclear region) is auto-fluorescence. TZM-bl cells infected with INmCherry-labeled HIV-1 were imaged starting at 16 hpi. The movie is a maximum intensity projection of 1 µm Z-slices. Scale bar is 5 µm.

File Name: Supplementary Movie 7
Description: **Addition of a high-dose PF74 results in rapid displacement of immobile VRCs in TZM-bl nucleus (Related to Fig. 5e, f).** Movie shows the lack of intranuclear trafficking of INmNG-labeled VRCs (green, marked by trajectory overlays) which become highly mobile after the addition of 25 µM PF74. The green fuzzy signal in the cytoplasm (perinuclear region) is autofluorescence. TZM-bl cells infected with INmNG-labeled HIV-1 were imaged beginning at 4 hpi at 2.5s/ volume for 30 min pre- and post-PF74 addition. Images were acquired using the Zeiss Airyscan imaging module, as described in methods. The single particle trajectory (+/- 5 frames) was overlaid onto the movie using ICY image software. Movie begins at 5 hpi and is a 1 µm maximum intensity projection of central Z-slices. Scale bar is 5 µm.
